# Fractionated Stereotactic Radiotherapy for Brain Metastases: A Moroccan Multicenter Experience

**DOI:** 10.7759/cureus.87531

**Published:** 2025-07-08

**Authors:** Fatima Safini, Zainab Oumouloud, Abdelkader Acharki, Issam Lalya, Nadia Benchakroun, Selma Kadiri, Mehdi El Ouartiti, Najib Derhem, Abdelmounaim Zayane, Abdenbi El Moutaoukkil, Khawla Lfara, Bouchra Amaoui

**Affiliations:** 1 Biomed Laboratory, Faculty of Medicine and Pharmacy, Ibn Zohr University, Agadir, MAR; 2 Radiation Therapy, Souss-Massa University Hospital Center, Agadir, MAR; 3 Radiation Oncology, Moroccan Radiotherapy Oncology Association (AORAM), Casablanca, MAR; 4 Radiation Oncology, Ryad Oncologia Clinic, Casablanca, MAR; 5 Radiation Oncology, Mohammed V Military Teaching Hospital, Rabat, MAR; 6 Radiation Oncology, Mohammed VI Oncology Center-Casablanca University Hospital, Casablanca, MAR; 7 Radiation Therapy, Nakhil Centre of Oncology, Rabat, MAR; 8 Radiation Oncology, Agadir International Hospital, Akdital, Agadir, MAR; 9 Radiation Oncology, Private Oncology Practice, Agadir, MAR; 10 Radiation Oncology, Cheikh Maelainin Clinic, Agadir, MAR; 11 Radiation Therapy, Cheikh Maelainin Clinic, Agadir, MAR; 12 Physics, Laboratory of High Energy Physics, Faculty of Sciences, Mohammed V University, Rabat, MAR; 13 Radiation Oncology, Atlantic Centre of Oncology, Agadir, MAR

**Keywords:** brain, fractionated, hypofractionated, metastases, stereotactic radiotherapy

## Abstract

Introduction: Stereotactic radiotherapy is currently an essential therapeutic tool in the treatment of brain metastases. The aim of our study was to report a multicenter Moroccan experience on stereotactic radiotherapy using linear accelerators in the treatment of brain metastases.

Material and methods: We conducted a multicenter retrospective study that included 130 patients treated at five centers in Morocco for brain metastases during the period between January 2021 and December 2024. All patients received fractionated stereotactic radiotherapy delivered by a linear accelerator. Statistical analysis of the data was performed using Jamovi version 2.3.

Results: We collected 130 patients with 248 treated brain metastases. The mean age was 58.4 (24-88) with a female predominance (59.2%). The most common primary tumor was lung cancer (77 patients, 59.2%), followed by breast cancer (29 patients, 22.3%) and colorectal cancer (eight patients, 6.1%). Adenocarcinoma was the predominant histological type (104 patients, 73.5%), followed by squamous cell carcinoma (11 patients, 8.5%). The majority of patients had fewer than three metastases. The mean tumor size was 23.8 mm, and the median volume was 8 cc (0.05-89). The two most prescribed irradiation regimens were 3 × 9 Gy and 5 × 6 Gy (37 cases or 28%). The lowest biologically effective dose with α/β = 10 (BED10) was 35.7 in five patients (4%), with a mean BED10 of 51. Follow-up data were available for 59 patients. After a median follow-up of 10.9 months (2-36 months) since the onset of brain metastases, overall survival at 24 months was 48.2%.

Conclusion: Stereotactic radiotherapy remains an effective treatment for local control of brain metastases. However, further studies are needed to homogenize practices and establish guidelines and procedures to improve long-term results.

## Introduction

Brain metastases (BM) continue to be a major significant cause of morbidity and mortality in patients with metastatic cancer. More than 100,000 persons are diagnosed annually, and approximately 10%-30% of cancer patients will develop BM during the course of their disease [1]. Cerebral stereotactic radiotherapy represents a major advance in the treatment of BM. This high-precision technique delivers high doses to the tumor while preserving the surrounding normal brain tissue, thus improving local control and quality of life for patients [2]. This radiotherapy can be performed either in a single fraction (stereotactic radiosurgery (SRS)) or in several fractions (fractionated stereotactic radiotherapy (FSRT)). In this study, we report on a multicenter Moroccan experience of stereotactic radiotherapy using a linear accelerator in the treatment of BM.

## Materials and methods

This retrospective multicenter study was conducted across five radiotherapy institutions in Morocco: Ryad Oncologia Clinic (Casablanca), Nakhil Centre of Oncology (Rabat), Agadir International Hospital, Akdital (Agadir), Cheikh Maelainin Clinic (Agadir), and Atlantic Centre of Oncology (Agadir). We included patients irradiated in these different centers during the period between January 2021 and December 2024. All patients received FSRT, whether with single or multiple BM. All patients were treated with a linear accelerator. We excluded from our study patients treated with a single fraction or those treated with standard fractionation. Data were collected from patients' clinical records using a common data collection form, while respecting anonymity. Statistical analysis was performed using Jamovi version 2.3 (2022). The results of the analysis of quantitative variables were expressed as means and standard deviations, and those of categorical variables as numbers and frequencies. Kaplan-Meier curves were used to express overall survival and survival after the occurrence of BM.

## Results

One hundred and thirty patients with 248 BM treated in the five centers during the years 2021 and 2024 were enrolled in this retrospective study. The mean age of our patients was 58.4 years (range 24-88 years). Male predominance was noted, with 78 men (59.2% of cases) and 53 women (40.8% of cases). As regards the primitive tumors, we noted a predominance of bronchopulmonary cancer (77 cases, 59.2%), followed by breast cancer (29 cases, 22.3%) and gastrointestinal cancer (10 cases, 7.7%). BM were metachronous in 67 patients, with a mean time to onset after diagnosis of the primitive tumor of 26 months. BM occurred in an oligometastatic context in 40 patients (67.8%). A summary of the characteristics of patients treated with FSRT is presented in Table 1.

**Table 1:** Characteristics of patients at the time of FSRT ADK: adenocarcinoma; SCC: squamous cell carcinoma; CCC: clear cell carcinoma; FSRT: fractionated stereotactic radiotherapy

Characteristics	Value
Patient age (years), median	58 (24-88)
Sex, n (%)	
Men	77 (59.2%)
Women	53 (40.8%)
Primary cancer, n (%)	
Lung cancer	77 (59.2%)
Breast cancer	29 (22.3%)
Gastrointestinal carcinoma	10 (7.7%)
Malignant melanoma	3 (2.3%)
Renal cell carcinoma	3 (2.3%)
Gynecological cancer	4 (3%)
Other	4 (3%)
Histology	
ADK	104 (73.5%)
SCC	11 (8.5%)
CCC	3 (2.3%)
Melanoma	3 (2.3%)
Neuroendocrine tumors	3 (2.3%)
Others	6 (4.6%)

The mean value of the maximum diameter of the lesions was 23.8 mm (4-63 mm). Majority of the lesions were smaller than 30 mm, with a median volume of 8 cc (0.05-89). Most patients were treated for a single BM (56.6%) or two metastases (20.9%), without prior brain radiotherapy in 93.1% of cases. For the planning target volume (PTV), a 2 mm margin was applied to the gross tumor volume (GTV) in the majority of cases, but other margins were used: 3 mm in 23% of cases and 1 mm in 16% of cases. The mono-isocentric technique was used in 84.9% of cases. Different dose fractionations were used. Radiation therapy was delivered in three fractions of 7-11 Gy in 89 cases (68.5%), followed by five fractions of 6-7 Gy in 41 cases (31.5%) with an average biologically effective dose with α/β = 10 (BED10) of 51 (Table 2).

**Table 2:** Tumor and treatment characteristics WBRT: whole-brain radiotherapy; FSRT: fractionated stereotactic radiotherapy; RS: radiosurgery

Characteristics	Value
WBRT before RS	
Yes	9 (6.9%)
No	121 (93.1%)
Metastasis surgery	
Yes	6 (4.6%)
No	124 (95.4%)
Systemic treatment	
Yes	31 (23.8%)
Missing/unknown	99 (76.2%)
Extracranial metastases, n (%)	
Yes	48 (65.8%)
No	25 (34.2%)
Missing/unknown	9 (12.3%)
Time of occurrence	
Synchronous	63 (48.5%)
Metachronous	67 (51.5%)
Number of metastases	
1	73 (56.6%)
2	27 (20.9%)
3	12 (9.3%)
4	12 (9.3%)
≥5	6 (3.9%)
Max diameter lesion (median value) (cm)	2.2 (0.4-6.3)
Lesion volume (median value) (cm^3^)	8 cc (0.05-89)
Radiation dose (Gy)	
3 × 7	5 (4%)
3 × 7.7	5 (4%)
3 × 8	17 (13%)
3 × 9	37 (28%)
3 × 10	21 (16%)
3 × 11	5 (4%)
5 × 6	37 (28%)
5 × 7	4 (3%)
Response of the metastases on 1st follow-up, n (%)	
Responsive disease	17 (13%)
Stable disease	18 (13.9%)
Progressive disease	24 (18.5%)
Missing/unknown	71 (54.6%)

**Figure 1:**
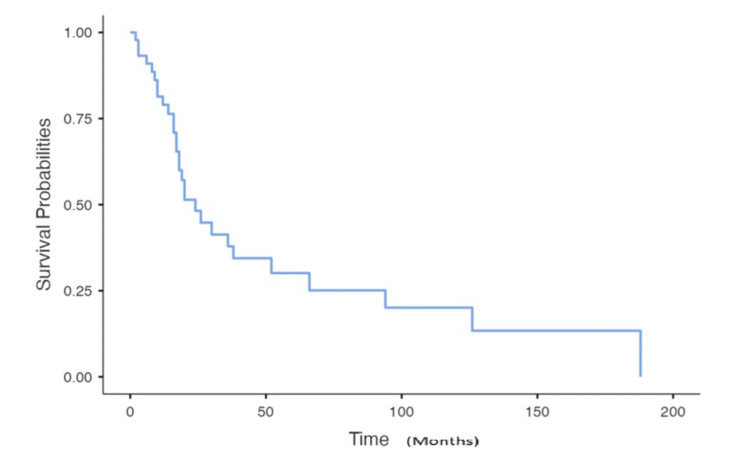
Overall survival according to Kaplan-Meier curves

**Figure 2:**
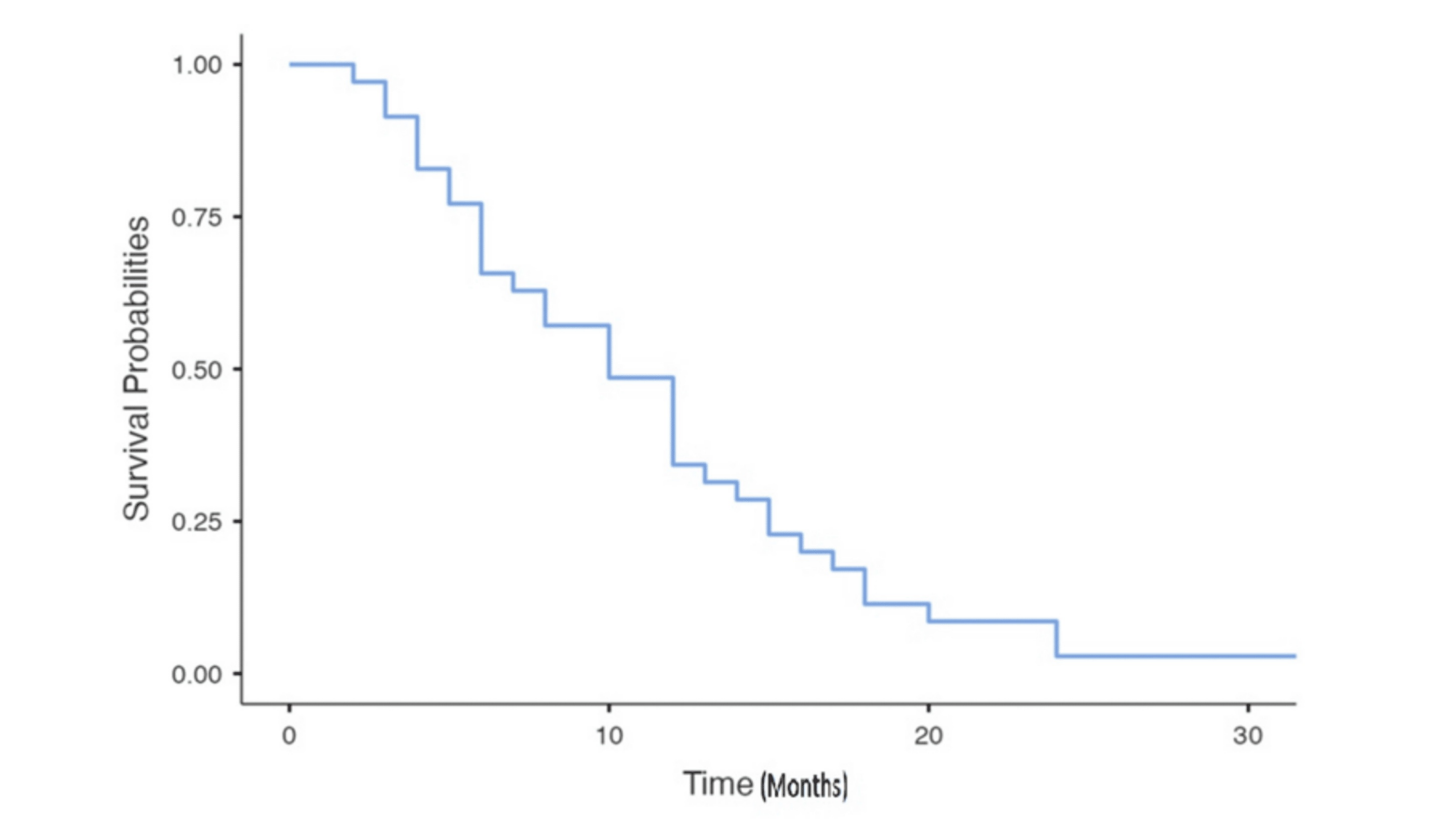
Survival after the occurrence of brain metastases

Follow-up data were available for only 59 patients. After a median follow-up of 10.9 months since the onset of BM, we observed stability in 18 patients, complete response in 17 patients, and progression in 24 patients. Overall survival at 24 months was 48.2%, with a median survival after BM of 10 months (Figures 1, 2).

## Discussion

Radiotherapy in Morocco has made giant technological strides, with 39 anti-cancer centers (public and private sectors) and over 70 linear accelerators at last count in 2023, making Morocco one of the best-equipped countries in Africa [3]. Although stereotactic radiation has been used in Morocco for over a decade, the practice has only become widespread in the various regions of the country in the last five years. The treatment of BM, initially based on whole-brain radiation therapy (WBRT), has benefited from technical advances in radiotherapy, especially stereotactic radiotherapy. This technique reduced neurocognitive complications and ensured good local control [4,5]. The choice between SRS (monofraction) and FSRT depends on the characteristics of the BM (number, size, and location). FSRT is generally preferred to SRS in cases of large BM, proximity to critical organs at risk (brainstem, chiasma, optic nerves, and hippocampus), or prior irradiation or in patients with comorbidities, especially vascular ones [6]. In our series, all patients received FSRT regardless of tumor size and location. More than half the metastases were less than 3 cm in diameter, and prior irradiation was reported in only nine patients (6.9%). The number of metastases treated with FSRT can be as high as 10 lesions. The prospective trial by Shuto et al. whose mature data were published in 2018 with a 60-month follow-up in 784 patients with BM from a pulmonary primary demonstrated that overall survival and side effects were the same in the groups with two to four and with five to 10 metastases [7]. The other parameter used to decide whether or not SRS is feasible is the total cumulative volume of BM expressed in mL or cm^3^. Various threshold values have been proposed in the literature: 7 cm^3^ (a sphere 2.4 cm in diameter) in the guidelines of the American Association of Neurosurgeons [8], 15 cm^3^ in the study by Yamamoto et al., and 30 cm^3^ in the Anocef guidelines (a sphere 3.9 cm in diameter) [9]. Margins around the GTV remain debatable and vary in the literature between no margin and 2 mm [10]. Kirkpatrick et al. randomly compared a GTV margin of 1 mm with 3 mm. They found no difference in terms of local control or occurrence of radionecrosis [11]. In our study, we observed a disparity in margins between the different centers.

FSRT has been used to reduce the risk of radionecrosis compared with single-fraction SRS. Lehrer et al.'s meta-analysis, which included 24 studies, attempted to compare local control and toxicity outcomes between these two fractionations for large-volume metastases. The authors concluded that hypofractionated stereotactic radiotherapy (HFSRT) maintains good local control while reducing the risk of radionecrosis [12]. FSRT delivers a high BED10 Gy while reducing the dose to organs at risk. Note that a low BED is associated with poor local control. The minimum BED10 Gy should be greater than 50 Gy, but a very high BED is associated with complications such as radionecrosis. The dose and protocols to be delivered in FSRT are not well codified. Numerous protocols and fractionations have been published in the literature, but treatment in three or five fractions remains the most widely used. The dose per fraction varies between 4 and 10 Gy in 10 to three fractions. The study by Wu et al., which aimed to establish a dose-response relationship between physical dose (BED) and local control in patients treated with FSRT using a linear accelerator, emphasized the importance of the mean dose and the dose received at the center of the PTV [13]. In our Moroccan context, we unfortunately do not have any guidelines for stereotactic radiotherapy of BM. The choice of dose and fractionation depends on the experience of the doctors and the habits of each center.

This study has many limitations. We were unable to obtain local control or toxicity data. Also, given the retrospective nature of the study, the limited sample size, and the use of different doses, correlation analyses to determine prognostic and predictive factors were not possible. We were unable to collect control imaging and toxicity data. In this study, we have no data on the systemic treatment received by patients, with disparities in practice between different centers (different PTV margins, dose, and fractionation).

## Conclusions

This multicenter study reports the first Moroccan experience with FSRT of BM using a linear accelerator. Although our study has a short follow-up time and few data on the evolution, it is an interesting experience that highlights the need to establish national recommendations in order to speak the same language, to have homogeneous data, and to develop national reference systems to improve our oncological results.
